# Emergence of a *tet*(M) Variant Conferring Resistance to Tigecycline in *Streptococcus suis*

**DOI:** 10.3389/fvets.2021.709327

**Published:** 2021-08-19

**Authors:** Rui Yu, Yue Zhang, Yindi Xu, Stefan Schwarz, Xin-Sheng Li, Yan-Hong Shang, Xiang-Dang Du

**Affiliations:** ^1^College of Veterinary Medicine, Henan Agricultural University, Zhengzhou, China; ^2^Institute for Animal Husbandry and Veterinary Research, Henan Academy of Agricultural Sciences, Zhengzhou, China; ^3^Department of Veterinary Medicine, Centre for Infection Medicine, Institute of Microbiology and Epizootics, Freie Universität Berlin, Berlin, Germany

**Keywords:** *Streptococcus suis*, genomic island, tigecycline, resistance, *tet*(M) variant

## Abstract

The aim of this study was to gain insight into the resistance determinants conferring resistance to tigecycline in *Streptococcus* (*S*.) *suis* and to investigate the genetic elements involved in their horizontal transfer. A total of 31 tetracycline-resistant *S. suis* isolates were screened for tigecycline resistance by broth microdilution. *S. suis* isolate SC128 was subjected to whole genome sequencing with particular reference to resistance determinants involved in tigecycline resistance. Transferability of genomic island (GI) GI*Ssu*SC128 was investigated by transformation. The roles of *tet*(L) or *tet*(M) in contributing to tigecycline resistance in *S. suis* were confirmed by transformation using different *tet*(L)- or *tet*(M)-carrying constructs. Only *S. suis* SC128 showed a tigecycline resistance phenotype. A *tet*(L)-*tet*(M) and *catA8* co-carrying GI*Ssu*SC128 was identified in this isolate. After transfer of the novel GI into a susceptible recipient, this recipient showed the same tigecycline resistance phenotype. Further transfer experiments with specific *tet*(L)- or *tet*(M)-carrying constructs confirmed that only *tet*(M), but not *tet*(L), contributes to resistance to tigecycline. Protein sequence analysis identified a Tet(M) variant, which is responsible for tigecycline resistance in *S. suis* SC128. It displayed 94.8% amino acid identity with the reference Tet(M) of *Enterococcus faecium* DO plasmid 1. To the best of our knowledge, this is the first time that a *tet*(M) variant conferring resistance to tigecycline was identified in *S. suis*. Its location on a GI will accelerate its transmission among the *S. suis* population.

## Introduction

Tigecycline, a semisynthetic antibiotic that binds to 16S rRNA and prevents translation of mRNA, is one of the last-resort antibiotics to treat complicated infections caused by multidrug-resistant Gram-negative and Gram-positive bacteria (1, 2). It belongs to the subgroup of tetracyclines called glycylcyclines (1). The presence of high-level tigecycline resistance genes, including several *tet*(X) variants such as *tet*(X3), *tet*(X4), *tet*(X6), and *tet*(X14), severely compromised the efficacy of tigecycline (3–8). In addition, several plasmid-mediated tetracycline resistance genes, such as variants of *tet*(A) and *tet*(L), have been reported to confer elevated tigecycline minimum inhibitory concentration (MICs) resulting in non-susceptibility (9, 10).

In production and breeding, the carrying rate of *S. suis* in pigs is as high as 80%. Despite a high carrier rate, morbidity rarely exceeds 5%, although it can reach more than 50% in cases of poor hygiene and concurrent disease (11). *S. suis* is also an important zoonotic pathogen. *S. suis* poses a threat to public health. In Vietnam, *S. suis* is the most important pathogen causing meningitis in adults (12). *S. suis* will not only cause huge economic losses to the breeding industry, but also have great harm to public health, especially the health of related practitioners.

In *S. suis*, the tetracycline resistance genes *tet*(O), *tet*(M), *tet*(W), *tet*(O/32/O), *tet*(S), *tet*(O/W/32/O), *tet*(40) and *tet*(B) have frequently been described (13–20). However, the tigecycline resistance mechanism in *S. suis* has not yet been explored.

In this study, the tetracycline resistance genes among *S. suis* isolates were investigated, with particular reference to the mechanism(s) involved in tigecycline resistance, and the genetic elements involved and their horizontal transfer.

## Materials and Methods

### Bacterial Strains and Plasmids

During 2011–2018, a total of 31 non-duplicate tetracycline-resistant *S. suis* strains were isolated and identified on Todd-Hewitt Agar (THA) plates containing 10 mg/L tetracycline from individual diseased pigs in four provinces (Henan, Shanxi, Shandong and Guangdong) in China. The *S. suis*-*Escherichia coli* shuttle plasmid vector pSET2s (SPE^+^) was used for cloning of the *tet*(L) gene. The serotype 2 strain *S. suis* P1/7 served as recipient strain in the transformation experiments. All *S. suis* strains were cultured in Todd-Hewitt Broth (THB) at 37°C.

### Antimicrobial Susceptibility Testing

Antimicrobial susceptibility testing (AST) was performed by broth microdilution according to the recommendations given by EUCAST and the results were categorized according to the EUCAST breakpoint tables for interpretation of MICs and zone diameters, Version 11.0 (21). *Streptococcus pneumoniae* ATCC 49619 served as the quality control strain.

### PCR Analyses

The *S. suis* isolates were investigated for the presence of the genes *tet*(K), *tet*(L), *tet*(M), *tet*(O), *tet*(Q), *tet*(T), *tet*(W), and *tet*(B) by PCR using the primers listed in Table 1 (22). The presence of a genomic island (GI) circular intermediate in *S. suis* SC128 and its transformant was detected by PCR using the primers circ_fw and circ_rv listed in Table 1.

**Table 1:** Oligonucleotide primer pairs used.

**Category and genes**	**Primer designation**	**Sequence (5′-3′)**	**Annealing tem (°C)**	**Product size (bp)**	**References**
*tet*(B)	*tet*(B)-fw *tet*(B)-rv	TTGGTTAGGGGCAAGTTTTG GTAATGGGCCAATAACACCG	55.0	659	(8)
*tet*(K)	*tet*(K)-fw *tet*(K)-rv	TCGATAGGAACAGCAGTA CAGCAGATCCTACTCCTT	52.0	169	(8)
*tet*(L)	*tet*(L)-fw *tet*(L)-rv	TCGTTAGCGTGCTGTCATTC GTATCCCACCAATGTAGCCG	54.0	267	(8)
*tet*(M)	*tet*(M)-fw *tet*(M)-rv	GTGGACAAAGGTACAACGAG CGGTAAAGTTCGTCACACAC	55.0	406	(8)
*tet*(O)	*tet*(O)-fw *tet*(O)-rv	AACTTAGGCATTCTGGCTCAC TCCCACTGTTCCATATCGTCA	57.0	515	(8)
*tet*(Q)	*tet*(Q)-fw *tet*(Q)-rv	TTATACTTCCTCCGGCATCG ATCGGTTCGAGAATGTCCAC	55.0	904	(8)
*tet*(T)	*tet*(T)-fw *tet*(T)-rv	AAGGTTTATTATATAAAAGTG AGGTGTATCTATGATATTTAC	46.0	169	(8)
*tet*(W)	*tet*(W)-fw *tet*(W)-rv	GAGAGCCTGCTATATGCCAGC GGGCGTATCCACAATGTTAAC	59.0	168	(8)
GI circle intermediate	circ_fw circ_rv	GACCCAACTAATACCTCTAA TAGTATCTGCCAAGTGAATG	47.3	976	This study
***tet*** **(L) introducing into** ***S. suis*** **P1/7**					
*tet*(L)-HA-up^a^	*tet*(L)-HA-up-fw^a^*tet*(L)-HA-up-rv^a^	TTCAGATTGTTCGGCTTGG GTCCGTTACACTAGAAAACCTTGGCTTCGGTGGTTCAT	56.5	948	This study
*tet*(L)_SC128_ used for transformation	*tet*(L)_SC128_-T-fw *tet*(L)_SC128_-T-rv	ATGAACCACCGAAGCCAAGGTTTTCTAGTGTAACGGAC GTCAATACAAACGCCTCCTTGTTAAACGAAACGAGATG	51.0	1,773	This study
*tet*(L)-HA-down^a^	*tet*(L)-HA-down-fw^a^*tet*(L)-HA-down-rv^a^	CATCTCGTTTCGTTTAACAAGGAGGCGTTTGTATTGAC GATAGAACCAAGCGGAAC	50.0	1,010	This study
***tet*** **(M) introducing into** ***S. suis*** **P1/7**					
*tet*(M)-HA-up^a^	*tet*(M)-HA-up-fw^a^*tet*(M)-HA-up-rv^a^	TTCAGATTGTTCGGCTTGG CGATATATGTTCAATAAAATAACTTAGTTGGCTTCGGTGGTTCAT	56.5	953	This study
*tet*(M)_SC128_	*tet*(M)_SC128_-fw *tet*(M)_SC128_-rv	ATGAACCACCGAAGCCAACTAAGTTATTTTATTGAACATATATC GGTCAATACAAACGCCTCCTTAGGAGGGCTTAGTTTTTTGTACC	52.7	2,136	This study
*tet*(M)-HA-down^a^	*tet*(M)-HA-down-fw^a^*tet*(M)-HA-down-rv^a^	GGTACAAAAAACTAAGCCCTCCTAAGGAGGCGTTTGTATTGAC GATAGAACCAAGCGGAAC	50.0	1,012	This study
***tet*** **(L) cloning into plasmid pSET2s**					
*tet*(L)_SC128_ used for cloning	*tet*(L)-C-fw *tet*(L)-C-rv	CGCGGATCCGGTTTTCTAGTGTAACGGAC CCCAAGCTTTTGTTAAACGAAACGAGATG	50.0	1,737	This study

a *HA, homologous arm*.

### Transformation Experiments

The transformation experiments were performed as described in previous studies (23, 24). The peptide (GNWGTWVEE) was used as a pheromone for the transformation. The detailed protocols for the transformation were as follows. The tetracycline-susceptible recipient strain *S. suis* P1/7 was grown to exponential phase at 37°C under 5% CO_2_. Then, the logarithmic P1/7 strains were diluted 1:50 into THY medium and grown at 37°C without shaking. When they reached an OD_600_ between 0.035 and 0.058, the chromosomal DNA (1.0 μg) of the donor *S. suis* SC128 and the synthetic peptide (250 μM) were added to 100 μL aliquots of the recipient strain. After 2 h, the samples were diluted, plated on THA plates containing 10 mg/L tetracycline, and incubated overnight at 37°C. Colonies were further confirmed by AST and multilocus sequence typing (MLST) following previous research (25).

### Whole Genome Sequencing and Protein Sequence Analysis

Whole genome DNA of *S. suis* SC128 was sequenced using the PacBio RS and Illumina MiSeq platforms (Shanghai Personal Biotechnology Co., Ltd., China). The PacBio sequence reads were assembled with HGAP4 and CANU (Version 1.6), and corrected by Illumina MiSeq with pilon (Version 1.22). The prediction of ORFs and their annotations were performed using Glimmer 3.0. Three methods were used to identify genomic islands as previously described, including GC content analysis (26, 27), whether it contains integrase (28), and PCR amplification of circular intermediates. Protein sequence analysis of the *tet*(M) gene was performed using the software DNAMAN 8.0.

### Introduction of the *tet*(L) and *tet*(M) Genes Into the Recipient by Homologous Recombination

In order to demonstrate the contribution of *tet*(L) or *tet*(M) located on GI*Ssu*SC128 to tigecycline resistance, *tet*(L)-carrying or *tet*(M)-carrying transformants were obtained by introduction of the *tet*(L) or the *tet*(M) genes into the tetracycline-susceptible *S. suis* P1/7, based on the protocol described previously (29). Briefly, as shown in Table 1, the two pairs of primers *tet*(L)-HA-up-fw/-rv and *tet*(L)-HA-down-fw/-rv were designed to amplify the upstream and downstream homologous fragments, which targets the glutamate dehydrogenase (GDH) gene and the dihydroorotate dehydrogenase (DHODH) gene from the recipient strain *S. suis* P1/7. The primers *tet*(L)_SC128_-T-fw/-rv were designed to amplify the intact copy of *tet*(L) including their promoter and translational attenuator from the donor strain *S. suis* SC128. The three amplification products were ligated by fusion PCR with primers *tet*(L)-HA-up-fw and *tet*(L)-HA-down-rv (30), and the recombination fragments were then introduced into *S. suis* P1/7 by transformation according to the method described in section “Transformation Experiments.” A concentration of 10 mg/L tetracycline was used for selecting the *tet*(L)-carrying transformants. In addition, the *tet*(M)-carrying transformants were generated in a similar way using the primers described in Table 1 and the same tetracycline concentration for selecting transformants.

### Functional Cloning of the *tet*(L) Gene

A 1,732 base pair (bp) fragment containing BamHI and HindIII restriction sites in the non-coding regions at the 5' and 3' termini of the *tet*(L) gene was amplified by PCR using a pair of primers *tet*(L)-C-fw/rv listed in Table 1. Then, the amplified fragment was ligated into the BamHI- and HindIII-digested *S. suis*-*E. coli* shuttle vector pSET2s. The recombinant plasmid pSET2s_*tet*(L) was then transformed into *E. coli* DH5α. After identification, the recombinant plasmid pSET2s_*tet*(L) was extracted and transformed into *S. suis* P1/7 by natural transformation as described above and the transformants were selected on the medium containing 100 mg/L of spectinomycin and grown at 37°C under 5% CO_2_ (23, 24).

## Results

### A Novel *tet*(L)-*tet*(M) Co-carrying GI Was Identified in *S. suis*

**Figure 1:**
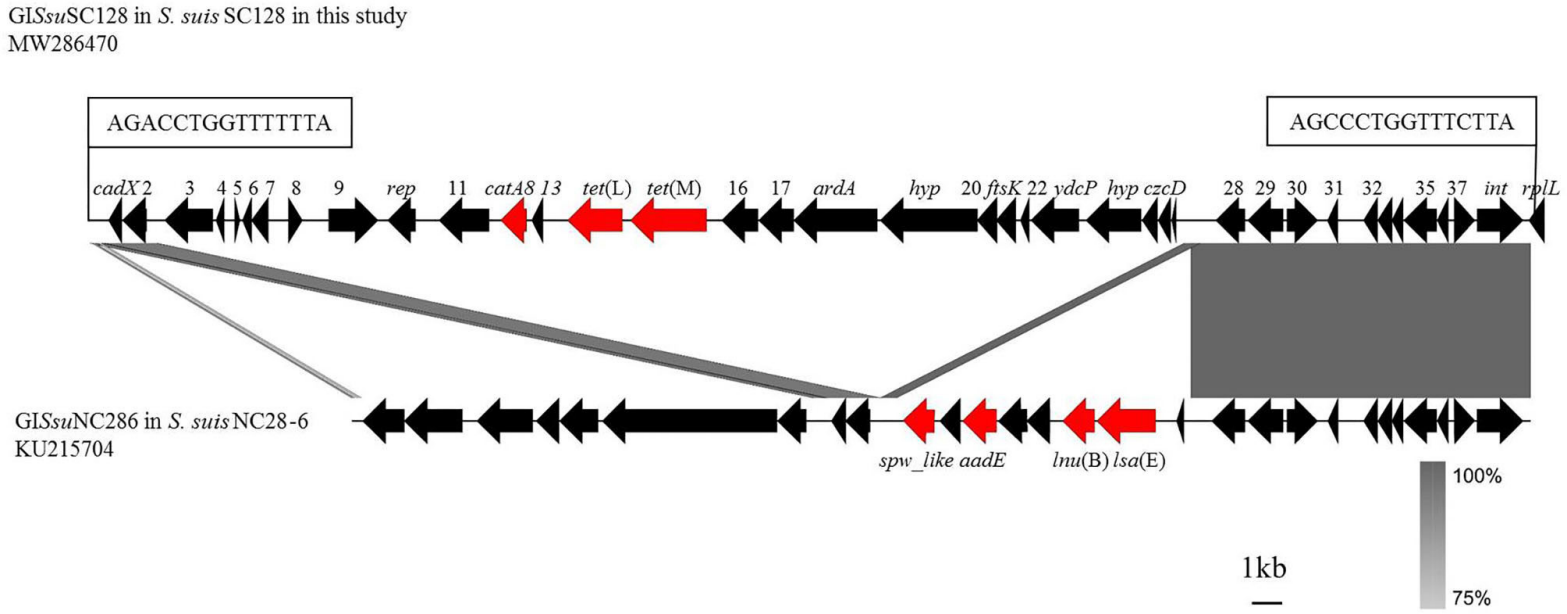
Comparison of GI*Ssu*SC128 in this study with GI*Ssu*NC286 in *S. suis* NC28-6 described previously. Analysis and creation of the image were performed by the software Easyfig2.2.3, the resistance genes are shown in red and other genes are shown in black. The locations of the PCR primers for the detection of circularizable forms of GI are indicated by arrows. The perfect 15 bp target site duplications at both termini are shown in boxes. Regions with more than 75% nucleotide sequence identity are shaded gray.

MIC results revealed that all 31 *S. suis* strains were tetracycline-resistant, which were further screened for the presence of tetracycline resistance genes using the primers listed in Table 1. Of them, *tet*(O) was detected in 30/31 of the total strains, with *tet*(M) in 1/30, and *tet*(L) in 1/30. Among them, the strain SC128 was positive for the combination of *tet*(L) and *tet*(M) and proved to be tigecycline resistant (Table 2). According to the interpretation criteria of MICs in the version 9.0 issued by EUCAST in 2021, *S. suis* is susceptible to tigecycline at a breakpoint of ≤0.125 mg/L, which means that a MIC > 0.125 mg/L is considered resistant (21). This strain was then selected for WGS. The GC percentage of SC128 was 41.03%, while that of the GI*Ssu*SC128 was 36.10%. GI*Ssu*SC128 contains an integrase. A circular intermediate was successfully amplified by PCR. These results indicated that GI*Ssu*SC128 was a genomic island. As shown in Figure 1, a novel *tet*(L)-*tet*(M) and *catA8* co-carrying GI was identified, designated ICE*Ssu*SC128, which has a size of 36.097 kbp. It was inserted at the *rplL* locus, which is one of the common insertion hotspots of mobile genetic elements (MGEs) in *S. suis*, forming 15 bp imperfect target site duplications at the integration site (5′-AGACCTGGTTTTTTA-3′ and 5′- AGCCCTGGTTTCTTA-3′) (31, 32). The DNA sequence of integrase in GI*Ssu*SC128 was compared to those deposited the NCBI GenBank, and the BLASTn result was that it had 100% identity and 100% coverage rate with the integrase in the *S. suis* NC28-6 genomic island. However, the 29.661 kbp GI*Ssu*NC286 in *S. suis* strain NC28-6 carried the resistance genes *spw*_like, *aadE, lnu*(B) and *lsa*(E), but not *tet*(L) or *tet*(M), which is present in GI*Ssu*SC128 (33).

**Table 2:** MICs of the *tet*(L)-*tet*(M) co-carrying *S. suis* strain, the recipient *S. suis* strain P1/7 and their transformants.

**Strains**	**MICs (mg/L)^a^**							
	**FFC**	**ERY**	**LIN**	**GEN**	**CHL**	**TIG**	**TET**	**SPE**
SC128	<1	128	64	64	16	0.25	32	16
P1/7	<1	<1	<1	2	<1	0.0625	<1	16
P1/7+GI*Ssu*SC128	<1	<1	<1	2	16	0.25	32	16
P1/7+*tet*(M)	<1	<1	<1	2	<1	0.25	32	16
P1/7+pSET2S	<1	<1	<1	2	<1	0.0625	<1	>512
P1/7+pSET2S_*tet*(L)	<1	<1	<1	2	<1	0.0625	16	>512

a *FFC, florfenicol; ERY, erythromycin; LIN, lincomycin; GEN, gentamicin; CHL, chloramphenicol; TIG, tigecycline; TET, tetracycline; SPE, spectinomycin*.

### The GI*Ssu*SC128 Is Transmissible

Using the primers and PCR conditions listed in Table 1, a 976 bp amplicon was detected, which suggested that GI*Ssu*SC128 has the ability to excise from the *S. suis* chromosomal DNA and to form a circular translocatable unit. Transferability of GI*Ssu*SC128 was investigated by natural transformation using *S. suis* P1/7 as the recipient. The transformant, designated P1/7+GI*Ssu*SC128, was successfully obtained. MICs for the *S. suis* strain SC128, the recipient strain *S. suis* P1/7, and the transformant P1/7+GI*Ssu*SC128 were shown in Table 2. The transformant P1/7-GI*Ssu*SC128 displayed a 4-fold increase in the MICs of tigecycline compared with the recipient strain *S. suis* P1/7.

### *tet*(M) but Not *tet*(L) in GI*Ssu*SC128 Confers Resistance to Tigecycline

To identify the individual role of either *tet*(L) or *tet*(M) alone in conferring resistance to tigecycline in *S. suis*, homologous recombination fragments were constructed, which consisted of the upstream and downstream homologous arm targeting the glutamate dehydrogenase (GDH) gene and the dihydroorotate dehydrogenase (DHODH) gene, respectively, from the recipient strain *S. suis* P1/7 and the intact copy of *tet*(L) or *tet*(M) including their putative regulatory regions from the donor strain *S. suis* SC128. These constructs were then transformed into *S. suis* P1/7 by homologous recombination. The *tet*(M)-carrying transformant was successfully obtained, and designated *S. suis* P1/7+*tet*(M). It displayed a 4-fold increase in the MICs of tigecycline compared with the parent strain *S. suis* P1/7 (Table 2), revealing that *tet*(M) in GI*Ssu*SC128 alone can confer resistance to tigecycline in *S. suis*. However, the introduction of *tet*(L) into *S. suis* P1/7 failed as no *tet*(L)-carrying transformants were obtained in three independent attempts.

To further eliminate the influence of experimental factors and to confirm the contributing role of *tet*(L) in GI*Ssu*SC128 to tigecycline resistance in *S. suis*, the *tet*(L) gene from GI*Ssu*SC128 was cloned into the plasmid vector pSET2s and then transformed into *S. suis* P1/7. The positive clone was designated *S. suis* P1/7+pSET2s_*tet*(L). MICs for *S. suis* P1/7, *S. suis* P1/7+pSET2s and *S. suis* P1/7+pSET2s_*tet*(L) were shown in Table 2. The transformant P1/7-GI*Ssu*SC128 displayed a 4-fold increase in the MICs of tigecycline, compared with the recipient strain *S. suis* P1/7, *S. suis* P1/7+pSET2s, and *S. suis* P1/7+pSET2s_*tet*(L), which which suggested *tet*(L) in GI*Ssu*SC128 has no influence on tigecycline resistance in *S. suis*. These data confirmed that it is *tet*(M), not *tet*(L), in GI*Ssu*SC128, which confers resistance to tigecycline in *S. suis* SC128.

### *tet*(M) in GI*Ssu*SC128 Is a Variant

**Figure 2:**
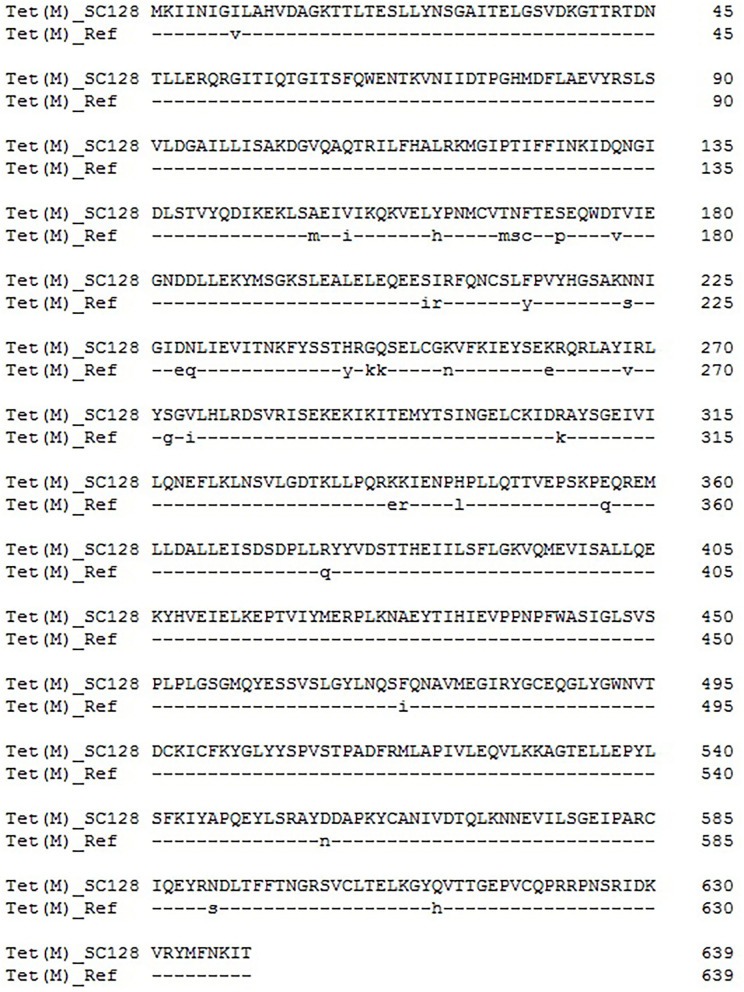
Amino acid substitutions occurring in the deduced amino acid sequence of the Tet(M) protein from *S. suis* SC128, compared with the reference Tet(M) from *E. faecium* DO plasmid 1 (accession number YP_006377310.1).

Previous studies showed that mutations in *tet*(M), which go along with deletions or amino acid substitutions, are associated with elevated tigecycline MICs, ranging from 0.0625 to 0.25 mg/L (34). In addition, a single deletion of L505 in the *tet*(M) caused the highest increase of tigecycline MIC (0.25 mg/L) (34). Protein sequence analysis with the software DNAMAN8.0 indicated that there are 33 amino acid substitutions occurring in the deduced amino acid sequence of the Tet(M) protein from *S. suis* SC128 (Identity, 94.8%), compared with the reference Tet(M) from *E. faecium* DO plasmid 1 (accession number YP_006377310.1) (Figure 2) (35). Thus, this GI-borne *tet*(M) gene is a naturally occurring variant.

## Discussion

Tigecycline has strong antibacterial activity against most Gram-positive and Gram-negative bacteria except *Proteus* and *Pseudomonas*, including *Enterobacteriaceae, Acinetobacter baumannii, Staphylococcus aureus, Enterococcus, Streptococcus pneumoniae*, etc. (36). Tigecycline has gradually become the most effective treatment for various bacterial infections among medical clinical antibacterial drugs due to its wide antibacterial spectrum, strong antibacterial activity, and low drug resistance rate, and it is also a last line of defense drug (37). However, with the continuous use of tigecycline in clinical anti-infective therapy, some researchers have isolated bacteria that are clinically resistant to tigecycline (38). The mechanism of bacterial resistance to tigecycline is very complex, mainly including the efflux pump mechanism, cell membrane pore channel protein variation, drug binding site changes and drug enzymatic degradation. Tet(M) is a ribosome protective protein, which prevents tetracycline from binding to 23S rRNA. Tet(L) is a member of the MFS efflux pump family, which can export tetracycline but is not effective against tigecycline (32).

This study confirmed that the *tet*(M) variant is located on the genomic island of a *S. suis* strain. Three methods were used to identify genomic islands (26–28), GC content analysis, integrase detection, and PCR amplification of circular intermediates. The results indicated that GI*Ssu*SC128 was a genomic island. Integrases are critical to genomic islands, because the genomic islands of *S. suis* are often classified according to the integrase sequence. The DNA sequence of integrase in the genomic island in this study was compared those deposited in the NCBI GenBank, and the BLASTn result was that it had 100% identity and 100% coverage rate with the integrase in the *S. suis* NC28-6 genomic island (33). However, the 29.661 kbp GI*Ssu*NC286 in *S. suis* strain NC28-6 carried the resistance genes *spw*_like, *aadE, lnu*(B) and *lsa*(E), but not *tet*(L) or *tet*(M), which are present in GI*Ssu*SC128. This genomic island can undergo horizontal transfer. After the transfer, the MIC value of tigecycline to the recipient bacteria increased 4-fold, which made the recipient bacteria resistant to tigecycline. One limitation in this study is that the EUCAST breakpoints of *Streptococcus* groups A, B, C, and G were used to determine tigecycline resistance in *S. suis*. Although these breakpoints are currently the most accurate reference for resistance determination in *S. suis* according to EUCAST guidelines, *S. suis* does not belong to these groups. Species-specific breakpoints according to EUCAST or similar guidelines must be determined before tigecycline resistance of *S. suis* isolate SC128 can be finally confirmed.

By comparing and analyzing the amino acid sequence of Tet(M) in this study with the reference Tet(M) sequence from *E. faecium* DO plasmid 1 (35), it is found that Tet(M) in this study has multiple mutations. The results can be used for future research and provide a basis for the key amino acid sites of Tet(M) conferring resistance to tigecycline. The amino acid sequence of Tet(M) was compared with those deposited in the NCBI GenBank, and the BLASTp result indicated that it had 100% identity and 100% coverage rate with that in an unpublished Lactobacillales strain (WP_024406177). Whole-genome sequencing revealed the genetic environment of *tet*(M) and the mechanism of horizontal transfer, and provided a theoretical basis for controlling the spread of drug-resistance genes.

## Conclusions

In this study, both the transformants P1/7+GI*Ssu*SC128 and P1/7+*tet*(M) displayed a tigecycline resistance phenotype. This observation indicated that *tet*(M) variants, whether they occurred naturally or were artificially induced, could confer resistance to tigecycline. The location of a *tet*(M) variant responsible for the tigecycline resistance on a GI will facilitate its transmission within the *S. suis* population.

## Data Availability Statement

The datasets presented in this study can be found in online repositories. The names of the repository/repositories and accession number(s) can be found below: https://www.ncbi.nlm.nih.gov/genbank/, MW286470.

## Author Contributions

Y-HS and X-DD designed the research and supervised the study. RY, YZ, YX, and X-SL performed the experiments and analyzed the data. RY, YZ, SS, and X-DD wrote the manuscript. All authors revised the manuscript and approved the final version for submission.

## Conflict of Interest

The authors declare that the research was conducted in the absence of any commercial or financial relationships that could be construed as a potential conflict of interest.

## Publisher's Note

All claims expressed in this article are solely those of the authors and do not necessarily represent those of their affiliated organizations, or those of the publisher, the editors and the reviewers. Any product that may be evaluated in this article, or claim that may be made by its manufacturer, is not guaranteed or endorsed by the publisher.
